# Distributed Learning: Revitalizing Anesthesiology Training in Resource-Limited Ethiopia

**DOI:** 10.3389/fpubh.2017.00059

**Published:** 2017-04-04

**Authors:** Krupa B. Patel, Morgan Dooley, Ananya Abate, Vanessa Moll

**Affiliations:** ^1^Department of Anesthesiology, Emory University School of Medicine, Atlanta, GA, USA; ^2^Department of Anesthesiology, Addis Ababa University School of Medicine, Addis Ababa, Ethiopia

**Keywords:** postgraduate medical education, resource-limited setting, e-learning, residency training, collaborative training, distance learning

## Abstract

**Background:**

Ethiopia has a significant paucity of available health-care workers. Despite the increasing number of medical schools, there are not enough physician instructors. Furthermore, availability and standardization of postgraduate training are lacking. Modalities of e-learning have been shown to be successful when used to impart medical education in other resource-limited countries. The Emory University and Addis Ababa University (AAU) Departments of Anesthesiology have formed a collaboration with the intent of improving the AAU Anesthesiology residency program, one of two postgraduate training programs for anesthesiology in Ethiopia.

**Methods:**

An initial educational needs assessment identified areas in the existing training program that required improvement. In this pilot study, we describe how the current classroom-based curriculum is augmented by the introduction of interactive educational sessions and distributed learning in the form of video lectures. Video lectures covered topics based on areas identified by Ethiopian residents and faculty. Interactive sessions included hands-on ultrasound workshops and epidural placement practicums, a journal club, problem-based learning sessions, and a mock code simulation. Assessment of the additions of the newly introduced blended learning technique was conducted *via* pre- and posttests on the topics presented.

**Results:**

Pre- to posttest score averages increased from 54.5% to 83.6%.

**Conclusion:**

An expansion of educational resources and modes of didactics are needed to fill the gaps that exist in Ethiopian anesthesiology training. Incorporating distributed learning into the existing didactic structure may lead to more efficacious instruction resulting in a higher retention rate of information.

## Introduction

Ethiopia is located in sub-Saharan Africa, with a population of 102.4 million, according to 2016 United Nations estimates (1, 2). The region has an insufficient number of health-care workers spanning all fields. The World Health Organization (WHO) recommends 23 health workers, including physicians, nurses, and midwives, per 10,000 people to achieve adequate primary care coverage (3, 4). This recommendation is in stark contrast to Ethiopia, which has approximately 1.8 health workers per 1,000 people, according to a 2009 WHO bulletin (4). There are 0.03 physicians per 1,000 people and specifically, 0.02 trained medical anesthetists per 100,000 people (3–5). An estimated 0.05% of physicians leave the workforce per year, while the inflow of medical school graduates to the workforce is estimated to be 0.075%. Despite the net increase in physicians, when coupled with the significant deficit and the rising population, it is clear that the shortage of physicians and other health-care workers will worsen (4). While there has been a rise in the establishment of medical schools in sub-Saharan Africa over the last two decades, the region still lags behind the rest of the world in the number of medical schools per capita. In 2014, East Africa had one medical school per 7.51 million people, one of the highest deficits in the world (6). In comparison, Ethiopia currently has 12 medical schools, with a ratio of 1 medical school for 8.5 million people (7). Additionally, there are still other challenges. For example, despite establishing medical schools, there are not enough physician instructors at the schools. Furthermore, there is a lack of standardization and availability of postgraduate training (8). To further augment the disparity of physicians, this lack of postgraduate training (residency) has been implicated as contributing to the migration of African-trained physicians to developed countries such as United States, United Kingdom, Canada, and Australia, sometimes referred to as the “brain drain” (5). The Addis Ababa University (AAU) Department of Anesthesiology has not been immune to this—between 1991 and 2003, 31 residents enrolled in the AAU Anesthesiology residency. Five residents dropped out during the course of residency, and two others have passed away after graduating. Of the remaining 24 graduates, 10, or 41.6% of graduates, migrated abroad and are currently practicing in other countries. Furthermore, the lack of fellowship opportunities drives African trainees to seek opportunities for further training abroad. Academic institutions from developed countries have been involved in developing postgraduate training programs in sub-Saharan Africa to both address the need for such programs and to hopefully stem the outflow of trainees seeking these opportunities elsewhere (5). Emory University School of Medicine has established a collaboration with the AAUSOM in 10 specialties and in 2016, the Emory Department of Anesthesiology joined the list as the 11th. The aim of this partnership is to augment the AAUSOM Anesthesiology residency program curriculum.

Modalities of e-learning have been shown to be successful when used to impart medical education in resource-limited countries, including sub-Saharan Africa (9). Using this precedent, we sought to introduce this concept of distributed learning, where learners and educators are separated by time and distance, to the AAUSOM Department of Anesthesiology (10). A blended learning technique, which is a combination of traditional classroom learning, where both the educators and trainees are present in person, and e-learning, in the form of video lectures, was employed (9, 10). Additionally, we introduced interactive sessions, such as journal clubs, problem-based learning sessions, ultrasound-guided skills/anatomy sessions, and mock code simulations to broaden the manner of instruction provided to residents from a strictly lecture-based approach.

## Methods

The AAUSOM Anesthesiology residency is a 3-year program that currently has 18 residents total—12 second-year residents and 6 first-year residents. The residents are exposed to a variety of surgical cases, including general surgery, trauma, ENT, neurosurgery, thoracic surgery, orthopedics, gynecology, pediatrics, and urology, in addition to experiences outside of the operating room, such as the intensive care unit, preoperative clinic, and obstetrics. The academic year is from January to December, and the educational curriculum currently consists of Wednesday seminar days, during which all 18 residents are excused from their duties to attend lectures. These lectures are provided by 1–2 residents per week, and the topics are preselected at the start of the academic year. The remainder of resident education is provided when working with attending physicians in the operating room, preoperative clinic, or during rounds in the intensive care unit.

A needs assessment was first conducted by communicating directly with AAUSOM Department of Anesthesiology faculty regarding their residency program, areas of weakness, and goals they had for our involvement. Additionally, an Emory Anesthesiology Global Health Residency Scholars Program (GHRSP) faculty member traveled to Addis Ababa, Ethiopia prior to our 1-month trip to conduct a site visit at the Black Lion Hospital and meet with the AAUSOM Department of Anesthesiology faculty, where possible educational interventions and areas of involvement were discussed.

We developed a lecture series that was modeled after the introductory lectures provided for new Emory anesthesiology residents at the start of the residency program. The lecture series comprised of 12 25–30 min lectures. Table 1 displays a list of the topics included in the video lecture series compared with the topics covered by Emory University Department of Anesthesiology’s introductory lecture series. Prior to traveling to Ethiopia, the entire lecture series was recorded as .mp4 files using AdobeConnect (Adobe, CA, USA), which provides a visual of both the presenter and lecture slides, which were designed on PowerPoint (Microsoft, WA, USA), complete with a pointer and live annotation, and audio. These lecture recordings were presented to the residents during the Wednesday seminar days—four lectures per seminar day, for a total of three Wednesday seminar days during the 1-month visit. The recordings were left with the residency program, loaded on to university computers and USB drives, to be used as an introductory lecture series for future incoming residents. Pretests and posttests were also developed, which consisted of three to four multiple choice and true/false questions per lecture. The pretests were administered immediately prior to presenting the lecture recordings. The posttests were administered one week later during the following Wednesday seminar day. We developed the video lecture series as a pilot study to evaluate the feasibility and educational benefits to Ethiopian residents when sending video lectures abroad without the lecturer present. We were awarded the Emory GHRSP Medical Education Mini-Grant prior to our departure, which allowed for the purchase of a projector that was used to present the lecture recordings and USB drives that were provided to each of the residents.

**Table 1:** **Topics covered by video lecture series and topics covered by Emory clinical anesthesia year 1 (CA-1) lecture series**.

Video lecture series	Emory CA-1 lecture series
Airway management	Anesthesia machine
Acid–base disorders	Airway management
Electrolyte abnormalities	Ethics
Autonomic nervous system	PACU and patient transport
Volatile anesthetics: minimum alveolar concentration	Common emergencies
Volatile anesthetics: effects on organ systems	Minimum alveolar concentration
Volatile anesthetics: uptake and distribution	Positioning
Induction agents	Induction/inhalational agents
Opioids	Infection control
Neuromuscular blockade	Muscle relaxants
Postoperative nausea and vomiting	Fluids and electrolytes
Malignant hyperthermia	Uptake and distribution
	Respiratory functions and pulmonary pathophysiology

In addition to our weekly lecture series, we provided daily interactive teaching sessions. Table 2 displays the covered topics. During our visit, groups of seven to eight residents were excused from their rotation duties for 1 week at a time to work with our team. During our needs assessment, we had identified that the residents did not have much experience with ultrasonography or regional anesthesia, including placement of lumbar labor epidurals. The focus of our first 2 weeks was ultrasonography. In addition to a portable ultrasound machine with a vascular probe that was provided by the AAUSOM Department of Anesthesiology, we brought an ultrasound machine with a cardiac, curvilinear, and linear probes for teaching purposes. The second 2 weeks focused on obstetrical anesthesia. In addition to ultrasound skills and obstetric anesthesiology, we also led a mock code simulation and presented a journal club to introduce the residents to critical evaluation of the medical literature.

**Table 2:** **Topics covered during daily interactive teaching sessions, both didactic and practical**.

Daily interactive teaching sessions	
Weeks 1–2: ultrasonography	Basics of ultrasonography Focused cardiac examination
Lectures and practical sessions	Lung ultrasound 3-point compression technique for lower extremity deep vein thrombosis detection Vascular access Upper and lower extremity peripheral nerve blocks (and relevant anatomy)
Weeks 3–4: obstetric anesthesiology	Maternal–fetal physiology Lumbar epidural catheter placement technique
Lectures, problem-based learning sessions, and practical sessions	Epidural catheter management and troubleshooting Use of epidural local anesthetics and narcotics Risks and adverse events associated with use of epidural catheters

Learning objectives included demonstrating an increase in knowledge in the lecture topics that comprised the video lecture series, demonstrated in Table 1, gaining familiarity with ultrasound and its applications to the practice of anesthesiology and critical care medicine, and increasing the depth of knowledge in obstetric anesthesiology (Table 2).

## Results

**Figure 1:**
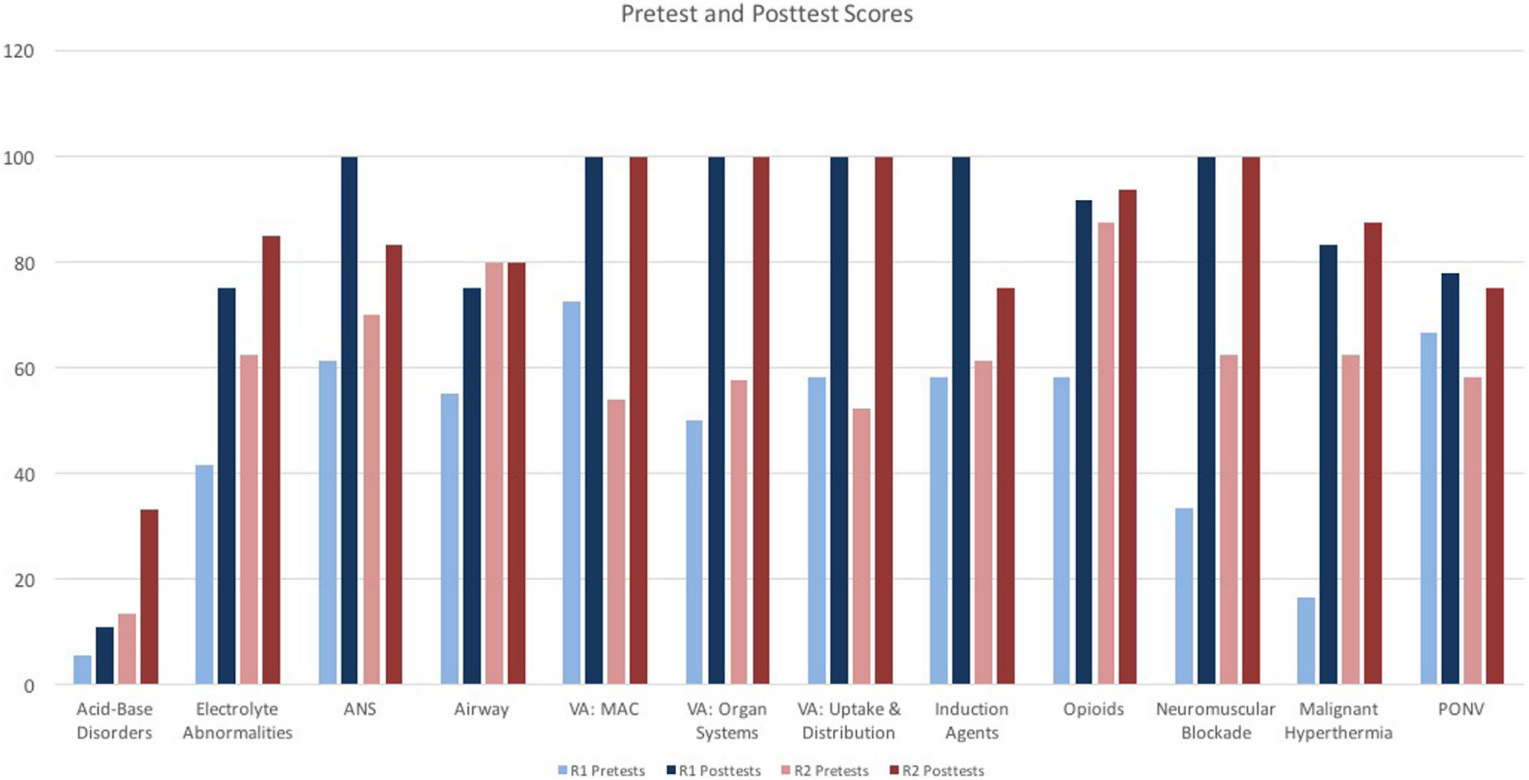
**Pretest and posttest scores**. ANS, autonomic nervous system; VA, volatile anesthetics; MAC, minimal alveolar concentration; PONV, postoperative nausea and vomiting.

Of the 18 residents in AAUSOM Anesthesiology residency program, 17 residents participated in this study by taking any of the 12 pre- and posttests. The pre- and posttest scores for the 17 study participants are shown in both Figure 1 and Table 3. As displayed on Table 3, the overall pretest to posttest average for the entire residency program improved from 54.5% to 83.6%. When the results were narrowed to the 7 residents that took all 12 pre- and posttests, the averages were similar, as shown on Table 4: pretest average 55.9% and posttest average 86.2%. That is, the pretest and posttest averages of the residents that took all 12 sets of tests did not differ appreciably from the residents who only took some of the tests. While the second-year residents out-performed their first-year peers on pretests, with averages of 60.14% and 48.12%, respectively, the first-year residents surprisingly averaged slightly higher on posttests, with averages of 84.50% compared to 82.64% for the second-year residents. Of the individual lecture topics, both first- and second-year residents demonstrated the most improvement on volatile anesthetics: MAC; volatile anesthetics: organ systems; volatile anesthetics: uptake and distribution; and neuromuscular blockade. As demonstrated on Table 3, pretest averages for these topics ranged from 47.92% to 63.21%, while posttest averages increased to 100%. Both first- and second-year residents fared the worst on acid–base disorder, with a pretest average of 13.30% and posttest average of 20.55%.

**Table 3:** **Pretest and posttest scores of the 17 residents that took at least 1 of the 12 sets of pretests and posttests**.

Residents	Acid–base disorders		Electrolyte abnormalities		ANS		Airway		VA: MAC		VA: organ systems		VA: uptake and distribution		Induction agents		Opioids		Neuromuscular blockade		Malignant hyperthermia		PONV		Overall	
	Pre	Post	Pre	Post	Pre	Post	Pre	Post	Pre	Post	Pre	Post	Pre	Post	Pre	Post	Pre	Post	Pre	Post	Pre	Post	Pre	Post	Pre	Post
**First year^a^**																										
1	0	0	50	75	67	100	75	75	67	100	33	100	75	100	75	100	75	100	50	100	25	100	33	67	52.1	84.8
2	0	0	50	50	100	100	25	50	67		67		50		50										51.1	50
3	33	33	50	100	33	100	75	100	67	100	33	100	25	100	50	100	25	75	25	100	0	50	67	67	40.3	85.4
4	0	0	25	75	33	100	50	100	100		67		50		50										46.9	68.8
5	0	0	25	75	67	100	50	50	67		67		75		50										50.1	56.3
6	0	33	50	75	67	100	55	75	67	100	33	100	75	100	75	100	75	100	25	100	25	100	100	100	53.9	90.3
Average	5.5	11	41.7	75	61.2	100	55	75	72.5	100	50	100	58.3	100	58.3	100	58.3	91.7	33.3	100	16.7	83.3	66.7	78	48.1	84.5
**Second year^a^**																										
1	0	100	75	75	33	100	100	100	25		33		100		100										58.3	93.8
2	0	0	75	75	67	67	75	75	0		67		25		75										48	54.3
3	33	33	50	75	100	100	75	50	100	100	67	100	75	100	50	100	75	75	75	100	75	100	33	33	67.3	80.5
4	0	33	50	75	100	100	50	100	33	100	33	100	50	100	50	100	75	100	25	100	50	100	33	67	45.8	89.6
5	0	33	75	100	67	100	75	75	33	100	67	100	75	100	75	50	100	100	50	100	50	50	100	100	63.9	84
6	0	0	0	100	33	33	100	100	67		67		50		50										45.9	58.3
7	0	33	50	75	33	67	75	50	67		100		25		50										50	56.3
8	33	33	100	75	100	67	100	100	67		0		25		75										62.5	68.8
9	67	33	75	100	67	100	75	75	67		67		25		50										61.6	77
10	0	33	75	100	100	100	75	75	67	100	33	100	50	100	75	50	100	100	100	100	75	100	67	100	68	88.2
11		0		25		0		25	67		100		75		25											12.5
Average	13.3	30.1	62.5	79.6	70	75.3	80	75	53.9	100	57.6	100	52.3	100	61.4	75	87.5	93.8	62.5	100	62.5	87.5	58.3	75	60.1	82.6
Overall average	13.3	20.6	52.1	77.3	65.6	87.9	67.5	75	63.2	100	53.8	100	55.3	100	59.9	87.5	72.9	92.7	47.9	100	39.6	85.4	62.5	76.5	54.5	83.6

*^a^Anonymous identifiers used for residents*.

*ANS, autonomic nervous system; VA, volatile anesthetics; MAC, minimal alveolar concentration; PONV, postoperative nausea and vomiting*.

**Table 4:** **Pretest and posttest scores of the 7 residents that took all 12 sets of pretests and posttests**.

Residents	Acid–base disorders		Electrolyte abnormalities		ANS		Airway		VA: MAC		VA: organ systems		VA: uptake and distribution		Induction agents		Opioids		Neuromuscular blockade		Malignant hyperthermia		PONV		Overall	
	Pre	Post	Pre	Post	Pre	Post	Pre	Post	Pre	Post	Pre	Post	Pre	Post	Pre	Post	Pre	Post	Pre	Post	Pre	Post	Pre	Post	Pre	Post
**First year^a^**																										
1	0	0	50	75	67	100	75	75	67	100	33	100	75	100	75	100	75	100	50	100	25	100	33	67	52.1	84.8
3	33	33	50	100	33	100	75	100	67	100	33	100	25	100	50	100	25	75	25	100	0	50	67	67	40.3	85.4
6	0	33	50	75	67	100	75	75	67	100	33	100	75	100	75	100	75	100	25	100	25	100	100	100	55.6	90.3
Average	11	22	50	83.3	55.7	100	75	83.3	67	100	33	100	58.3	100	66.7	100	58.3	91.7	33.3	100	16.7	83.3	66.7	78	49.3	86.8
**Second year^a^**																										
3	33	33	50	75	100	100	75	50	100	100	67	100	75	100	50	100	75	75	75	100	75	100	33	33	67.3	80.5
4	0	33	50	75	100	100	50	100	33	100	33	100	50	100	50	100	75	100	25	100	50	100	33	67	45.8	89.6
5	0	33	75	100	67	100	75	75	33	100	67	100	75	100	75	50	100	100	50	100	50	50	100	100	63.9	84
10	0	33	75	100	100	100	75	75	67	100	33	100	50	100	75	50	100	100	100	100	75	100	67	100	68.1	88.2
Average	8.3	33	62.5	87.5	91.8	100	68.8	75	58.3	100	50	100	62.5	100	62.5	75	87.5	93.8	62.5	100	62.5	87.5	58.3	75	61.3	85.6
Overall average	9.6	27.5	56.3	85.4	73.7	100	71.9	79.2	62.6	100	41.5	100	60.4	100	64.6	87.5	72.9	92.7	47.9	100	39.6	85.4	62.5	76.5	55.9	86.2

*^a^Anonymous identifiers used for residents*.

*ANS, autonomic nervous system; VA, volatile anesthetics; MAC, minimal alveolar concentration; PONV, postoperative nausea and vomiting*.

## Discussion

This study suggests that recorded video lectures resulted in an increase in knowledge, as demonstrated by improved test scores, in a group of first- and second-year anesthesiology residents in Ethiopia. This study is innovative in that it is the first study, to our knowledge, that applies use of video lectures to anesthesiology education in Africa. It is also the first study to evaluate methods to improve the specific knowledge gaps identified by the AAUSOM Anesthesiology residency.

There are multiple benefits of utilizing recorded lectures to augment medical or postgraduate residency education in a resource-limited setting. All residents have the opportunity to have a uniform educational experience. These lectures can be viewed at any time; being on-call or post-call, having an off-site rotation, or time off from work does not preclude a resident from attending lecture. Additionally, non-traditional students or residents who have responsibilities such as families or other jobs are able to view the lectures at their leisure. Recorded lectures can also be viewed multiple times and can be revisited prior to examinations as needed. Video lectures decrease the burden on a limited number of faculty members, who have clinical responsibilities that may overshadow educating residents. They can provide quality education in places that have few experienced, effective educators. Residents and students can be provided with an educational experience similar to what can be found in developed nations that likely practice in accordance to current practice guidelines, and use innovative medications and practice styles to which residents or students in developing countries may not be exposed. This would provide an experience that is elevated from that provided by the home program. This can result in improved training and the provision of better health care (11). Additionally, Ethiopian anesthesiology residents, for example, are expected to sit for European-style board examinations, despite the fact that many of the medications and practice styles are not available at their training sites and would benefit from video lectures providing this information. Recorded lectures also make global health initiatives more accessible for physicians in developed nations. Having the ability to remotely provide education in a resource-limited area eliminates the necessity to be physically present, thereby forgoing the cost, travel, and time away from work and family that need to be coordinated for traditional global health trips. This may increase interest and participation in global health ventures in developed countries.

There are many studies that show the benefits of distributed learning in the form of video lectures or video conferencing providing medical education. Schreiber et al. performed a randomized controlled trial investigating live lectures versus video podcasts in a population of first-year medical students in the United Kingdom. Knowledge-based assessments obtained in the form of multiple choice questions showed no statistical difference between the two groups (12). In a population of United States medical students, Topale demonstrated that use of recorded lectures did not diminish students’ participation in live lectures. Additionally, survey results indicated that given the demanding lifestyle during residency training, video lectures were a useful study aid and were frequently paired with other resources for revision and preparation for examinations (13). In a case–control study based in Uganda, Autry et al. showed that teaching surgical skills, such as knot tying, *via* videoconferencing was more efficacious than the usual practice of a 1-week skills course in a group of obstetrics and gynecology interns. Seventy-five percent of interns in the intervention arm had a score improvement of 50% or more, compared to 14% of the control group (14).

It should be stressed, however, that video lectures are efficacious at reinforcing and reviewing topics, but are not meant to replace traditional teaching methods. This is evidenced by the minimal rise in pretest to posttest scores for the acid–base disorders lecture topic. This topic is not taught as part of the AAU Anesthesiology residency curriculum. In fact, some residents reported that they last saw this topic years prior in medical school, while some residents did not recall ever receiving instruction on acid–base disorders. This likely results from the infrequent use of arterial blood gases perioperatively at their institution. This is in sharp contrast to the remainder of the topics covered in this lecture series that are also taught as part of the AAU Anesthesiology residency program; these other topics demonstrated more significant increases in pretest to posttest scores. In the study by Schreiber et al referenced above, despite equivalent test scores in both the live lecture and video lecture groups and despite video podcasts evaluated highly for convenience, overall satisfaction scores were lower for the video podcasts (12). This shows that while the use of technology is certainly a useful adjunct, it should not be used as the primary means of education.

There are limitations to recorded video lectures, the first being technology. AAUSOM’s teaching hospital, the Black Lion Hospital, where the anesthesiology resident lectures take place, has no reliable internet access. While we brought recorded lectures loaded onto USB drives when we physically traveled to Ethiopia, transmitting these lectures electronically is problematic. Nearly all of the AAU anesthesiology residents had access to a personal computing device. Some had access to the internet, either in the form of a smart phone or internet connection at home, therefore viewing electronically transmitted lectures from home or downloading them and sharing with classmates is a possibility. In general, the technology limitation in Ethiopia is not access to hardware, it is access to internet. A possible solution to this problem could be to send USB drives or CDs containing lectures *via* traditional postal service. While access to personal computing devices is not a grave limitation in Ethiopia, this does not hold true for all resource-limited areas. In other areas where these personal devices are more scarce than what we experienced in Ethiopia, it may be prudent to provide the residency program or the individual residents with a tablet that contains the recorded lectures.

Another limitation is the lack of open-ended communication. Should questions arise while viewing a recorded lecture, the resident would not have an instructor immediately present for assistance. Possible solutions include establishing a faculty member at the home program that serves as a point of contact for all inquiries regarding the video lectures. Another option would be an online forum or message board that would allow residents in developing nations to post questions, which could be answered remotely from instructors in other countries. This, again, is limited by the availability of internet and personal computing devices.

Limitations to our study include a possible confounder in the form of studying prior to administration of the posttests. Since the posttests were given 1 week after the lectures, motivated residents could study the lecture topics on their own time. Thus, the increase in pretest to posttest scores could have resulted from individual studying, not solely from the video lectures. Additionally, we did have daily interactive sessions with the residents in between the weekly video lecture sessions. While the topics of the interactive sessions, as displayed on Table 2, do not correlate with the topics of the video lectures, as displayed on Table 1, it is certainly possible that during an interactive session, residents acquired information that could have aided them in improving their posttest scores. Thus, because we were unable to control for resident independent studying and the content of our interactive sessions, it is conceivable that the video lectures were not solely responsible for the increase in posttest scores. Additionally, because we were limited by our 1-month trip, the posttests were administered 1 week after lectures, and we were unable to comment on long-term retention of information conveyed by the video lectures. A further limitation is our sample size. Of the 18 residents in the AAUSOM Anesthesiology residency program, 17 were included in this study. Unfortunately, not all 17 residents attended each lecture and took each pre- and posttest. Only seven residents met these criteria and were able to provide reliable data for the full duration of the study. While our study is underpowered and does not meet the standards for statistical significance, the general trend is promising. Additionally, this study only focuses on use of video lectures to improve knowledge gaps in anesthesiology residents; this limitation prevents us from generalizing our findings to other medical specialties. Future work can be focused on obtaining a larger sample size with longer follow-up to assess long-term retention, in addition to applying the same strategy to other medical specialty training programs to evaluate whether similar trends are obtained in non-anesthesiology residents.

This study could have been improved by better organization at the start of our month in Ethiopia; this could have resulted in optimal scheduling to allow all 18 residents to participate. Additionally, we could have arranged with the AAUSOM Department of Anesthesiology faculty to administer posttests at longer time intervals, such as 1 and 6 months, after our departure to assess long-term retention.

## Conclusion

This study demonstrates that the potential for recorded video lectures to augment traditional anesthesiology postgraduate training in resource-limited areas. Further work is needed to assess applications to medical education and postgraduate training for other specialties.

## Author Contributions

KP designed the project, which involved writing the 12 lectures in the lecture series and recording them, and writing pretest and posttest questions. She traveled to Ethiopia in June 2016 for 1 month to carry out the project and assist in the daily interactive teaching objectives. Upon returning to the United States, she analyzed the results and conducted a literature review, which culminated in writing this manuscript. MD supervised project design, traveled to Ethiopia for the latter 2 weeks of the trip, and supervised the daily interactive teaching sessions in obstetric anesthesiology. She proofread the manuscript. AA assisted with the needs assessment that led to our project design. He was the main contact on site in Ethiopia during the 1-month visit. He proofread the manuscript. VM supervised project design, traveled to Ethiopia for the first 2 weeks of the trip, and supervised the daily interactive teaching sessions in ultrasound skills and regional blocks. She proofread the manuscript.

## Conflict of Interest Statement

The authors declare that the research was conducted in the absence of any commercial or financial relationships that could be construed as a potential conflict of interest.

## References

[1] United Nations Populations Division. Worldometers. (2016). Available from: http://www.worldometers.info/world-population/ethiopia-population/

[2] World Health Organization. Global Health Observatory Data Repository: Ethiopia. (2014). Available from: http://www.who.int/gho/countries/eth/en/

[3] African Health Workforce Observatory. Human Resources for Health Country Profile: Ethiopia. (2010). Available from: http://www.hrh-observatory.afro.who.int/en/hrh-country-profiles/profile-by-country.html/

[4] Kinfu Y Dal Poz MR Mercer H Evans DB. The health worker shortage in Africa: are enough physicians and nurses being trained? Bull World Health Organ (2009) 87(3):225–30. 10.2471/BLT.08.051599 19377719 PMC2654639

[5] Walker I Pro-con debate. Con: pediatric anesthesia training in developing countries is best achieved by out of country scholarships. Pediatr Anesth (2009) 19:45–9. 10.1111/j.1460-9592.2008.02844.x 19076504

[6] Duvivier RJ Boulet JR Opalek A van Zanten M Norcini J Overview of the world’s medical schools: an update. Med Educ (2014) 48(9):860–9. 10.1111/medu.12499 25113113

[7] World Federation for Medical Education, Foundation for Advancement of International Medical Education and Research. World Directory of Medical Schools. (2016). Available from: http://wdoms.org

[8] Greysen SR Dovlo D Olapade-Olaopa EO Jacobs M Sewankambo N Mullan F Medical education in sub-Saharan Africa: a literature review. Med Educ (2011) 45:973–86. 10.1111/j.1365-2923.2011.04039.x 21916938

[9] Frehywot S Vovides Y Talib Z Mikhail N Ross J Wohltjen H E-learning in medical education in resource constrained low- and middle-income countries. Hum Resour Health (2013) 11:1–15. 10.1186/1478-4491-11-4 23379467 PMC3584907

[10] Oblinger DG Maruyama MK Distributed Learning, Vol. 14 Boulder, CO: CAUSE (1996). p. 1–28.

[11] O’Donovan J Ahn R Nelson BD Kagan C Burke TF. Using low-cost Android tablets and instructional videos to teach clinical skills to medical students in Kenya: a prospective study. JRSM Open (2016) 7(8):1–7. 10.1177/2054270416645044 27540487 PMC4973399

[12] Schreiber BE Fukuta J Gordon F Live lecture versus video podcast in undergraduate medical education: a randomized controlled trial. BMC Med Educ (2010) 10:68 10.1186/1472-6920-10-68 20932302 PMC2958969

[13] Topale L. The strategic use of lecture recordings to facilitate an active and self-directed learning approach. BMC Med Educ (2016) 16:201. 10.1186/s12909-016-0723-0 27520704 PMC4983083

[14] Autry A Knight S Lester F Dubowitz G Byamugisha J Nsubuga Y Teaching surgical skills using video internet communication in a resource-limited setting. Obstet Gynecol (2013) 122(1):127–31. 10.1097/AOG.0b013e3182964b8c 23743458

